# Unravelling the Effects of Syndecan-4 Knockdown on Skeletal Muscle Functions

**DOI:** 10.3390/ijms24086933

**Published:** 2023-04-08

**Authors:** Mónika Sztretye, Zoltán Singlár, Nyamkhuu Ganbat, Dána Al-Gaadi, Kitti Szabó, Zoltán Márton Köhler, László Dux, Anikó Keller-Pintér, László Csernoch, Péter Szentesi

**Affiliations:** 1 Department of Physiology, Faculty of Medicine, University of Debrecen, 4032 Debrecen, Hungary; 2 ELKH-DE Cell Physiology Research Group, 4032 Debrecen, Hungary; 3 Doctoral School of Molecular Medicine, University of Debrecen, 4032 Debrecen, Hungary; 4 Department of Biochemistry, Albert Szent-Györgyi Medical School, University of Szeged, 6720 Szeged, Hungary

**Keywords:** Syndecan-4, skeletal muscle, force, calcium homeostasis, aging

## Abstract

The remodelling of the extracellular matrix plays an important role in skeletal muscle development and regeneration. Syndecan-4 is a cell surface proteoglycan crucial for muscle differentiation. Syndecan-4^−/−^ mice have been reported to be unable to regenerate following muscle damage. To investigate the consequences of the decreased expression of Syndecan-4, we have studied the in vivo and in vitro muscle performance and the excitation–contraction coupling machinery in young and aged Syndecan-4^+/−^ (SDC4) mice. In vivo grip force was decreased significantly as well as the average and maximal speed of voluntary running in SDC4 mice, regardless of their age. The maximal in vitro twitch force was reduced in both EDL and soleus muscles from young and aged SDC4 mice. Ca^2+^ release from the sarcoplasmic reticulum decreased significantly in the FDB fibres of young SDC4 mice, while its voltage dependence was unchanged regardless of age. These findings were present in muscles from young and aged mice as well. On C2C12 murine skeletal muscle cells, we have also found altered calcium homeostasis upon Syndecan-4 silencing. The decreased expression of Syndecan-4 leads to reduced skeletal muscle performance in mice and altered motility in C2C12 myoblasts via altered calcium homeostasis. The altered muscle force performance develops at an early age and is maintained throughout the life course of the animal until old age.

## 1. Introduction

Skeletal muscles have great plasticity to answer to physiological challenges during maturation and exercise [1]. They can recover partly or totally from micro and macro injuries caused by exercise or other damages. Muscles contain cells (called myofibres or fibres) organized in bundles, and the extracellular matrix surrounds them both. The latter is composed of collagens, glycoproteins, and proteoglycans. The composition of the extracellular matrix is also highly plastic and helps in the adaptation of skeletal muscle to environmental effects during development, exercise, and regeneration. It stores and presents diverse cytokines and appropriate growth factors during muscle maturation [2].

Heparan sulphate (HS) proteoglycans are present at the cell surface and in the extracellular matrix. In mammalian cells, a four-member family of transmembrane proteoglycans with HS chains is termed syndecans (Syndecan-1 to 4), but only one of them, Syndecan-4, is expressed ubiquitously [3]. Syndecans were found to have a crucial role in muscle development, preservation, and regeneration, and were described to have widespread biological functions, including calcium channel regulation, cell adhesions, and migration [4,5,6]. Syndecans have a huge extracellular domain containing glycosaminoglycan binding sites, a highly conserved transmembrane domain, and a small intracellular domain, which is different in all syndecans. They also serve as co-receptors and transmit signals from the extracellular environment into the cells via their cytoplasmic domains [3]. It has been shown that Syndecan-4 can regulate the level of intracellular Ca^2+^ in different cell types [7,8,9]. Additionally, the Syndecan-4 level and its localization were acknowledged to be regulated by the electrical activity of the cells, suggesting a controlling mechanism influencing the adhesion of skeletal myotubes during differentiation [10]. An interesting finding was that all syndecans are present in muscles during development [4,11,12] and also in proliferating myoblasts, but their expression level decreases with time during the muscle maturation process [13,14]. Thus, its expression is high in myoblasts and young myotubes at the embryonic stage, but its amount starts to decline in myotubes after birth and it is present only in satellite cells in adults [4]. Syndecan-4 KO mice are incapable of regenerating following muscle injury and show impaired activation, proliferation, and differentiation of satellite cells [15]. These phenomena were accompanied by a thinner basal lamina of myofibres, suggesting a potential malfunction in the biosynthesis and turnover of the extracellular matrix [15]. Recent studies showed that Syndecan-4 KO mice had decreased muscle weight, thinner muscle fibres, and a decreased expression of transcription factors participating in myogenesis [16]. All of these findings raise the possibility that the absence of Syndecan-4 may also affect the proper function of skeletal muscles.

The development of muscle force includes a strict sequence of processes called the excitation–contraction coupling (ECC) (for a review see, e.g., [17,18]). The process starts with the arrival of the action potential from the motor neuron, which travels along the sarcolemma and reaches the inner part of the muscle fibre following the membranes of the transversal tubule (T-tubule). It initiates Ca^2+^ release from the intracellular store (sarcoplasmic reticulum, SR) by opening the calcium release channels (ryanodine receptor type 1, RyR1). The activation of RyR1 is triggered by a direct interaction with the L-type calcium channel (voltage sensor) situated in the T-tubule membrane (dihydropyridine receptor, DHPR). The released Ca^2+^ diffuses freely in the cytoplasm, reaches its binding site on the troponin-C (TnC), and the muscle then starts to contract. During relaxation, the SR calcium pump (SERCA) moves calcium released from the TnC back into the internal store. The formation of this very special structural organization and the direct coupling between RyR1 and DHPR is controlled by highly specific transcription and growth factors, and Syndecan-4 could have an impact on these.

Since previous studies demonstrated that syndecans 1–4 were differentially expressed in both embryonic tissue and satellite cells, [5,11] and their level showed an age-dependent decline and more specifically Syndecan-4 is present only in satellite cells in adult skeletal muscle fibres [19], we propose a crucial role of Syndecan-4 in muscle development, proper function, and in regeneration upon injury. As mature muscle contraction is constantly associated with micro-injuries throughout the whole lifetime of the individual and the regeneration capacity of the muscle decreases with age, the raised question is obvious: what is (if any) the role of syndecans in these processes?

The roles of Syndecan-4 in appropriate skeletal muscle function, force generation, and calcium homeostasis are still elusive. The description of its effects on the excitation–contraction coupling processes is totally missing. To shed light on these roles, we have studied the effects of Syndecan-4 ablation in fast and slow muscles from young and aged Syndecan-4^+/−^ (SDC4) mice and also on murine C2C12 myotubes. We have also analyzed the force development in vivo and in vitro, and the calcium homeostasis of developing and mature muscle fibres.

Based on our results we propose a role for Syndecan-4 in maintaining proper force generation and muscle performance both in young and aged animals as well as a role in maintaining calcium homeostasis in skeletal muscle and C2C12 myotubes.

## 2. Results

### 2.1. In Vivo Experiments

#### Decreased Grip Force and Voluntary Running in SDC4 Mice

Since the complete lack of Syndecan-4 in mice was shown to alter skeletal muscle structure [16], we aimed to investigate the effects of Syndecan-4 downregulation on overall muscle performance. Thus, we measured the body weight and assessed the in vivo physical performance of Syndecan-4^+/−^ (SDC4) mice. To study the upper body and overall strength, we measured forepaw grip force in young and aged mice from SDC4 and control (CTRL) animal groups. The maximal force of SDC4 animals was significantly smaller than that of CTRL animals (Table 1) in both age groups. The average body weight was also smaller in both SDC4 groups when compared with the age-matched CTRL mice. Furthermore, the normalized grip force remained significantly smaller in SDC4 animals (Table 1).

The voluntary running capability of mice was also investigated. Young mice in both groups conveyed almost the same distance daily; however, the SDC4 animals ran significantly slower but stayed longer in the running wheel than the CTRL animals. In contrast, the aged SDC4 mice spent significantly less time and thus covered less distance than the CTRL mice. The significant reduction in average and maximal velocity found in aged SDC4 mice was similar to that found in young animals (Table 2).

### 2.2. In Vitro Experiments

#### 2.2.1. Decreased Twitch Force in SDC4 Mice

**Figure 1:**
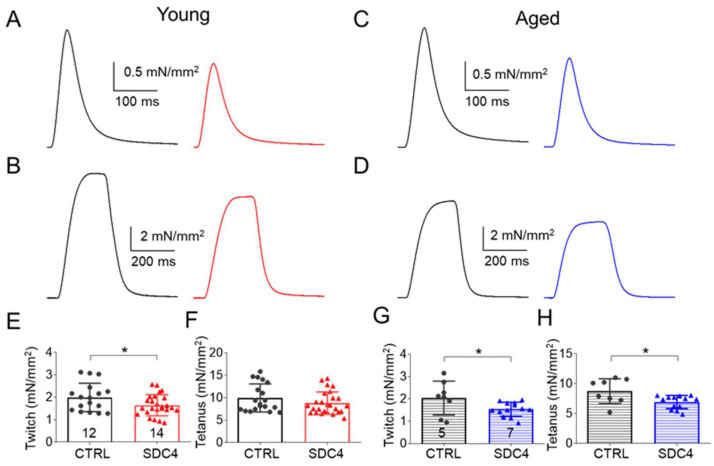
Representative ex vivo twitch (**A**,**C**) and tetanic (**B**,**D**) force on EDL muscle from young (**A**,**B**) and aged (**C**,**D**) CTRL (black) and SDC4 (red, blue) mice stimulated at 1 or 200 Hz, respectively, at room temperature (24 °C). Average twitch (**E**,**G**) and tetanic (**F**,**H**) force normalized to the cross-sectional area of the muscle. The numbers in the columns indicate the number of animals studied. * shows a significant difference from the age-matched CTRL at *p* < 0.05.

**Figure 2:**
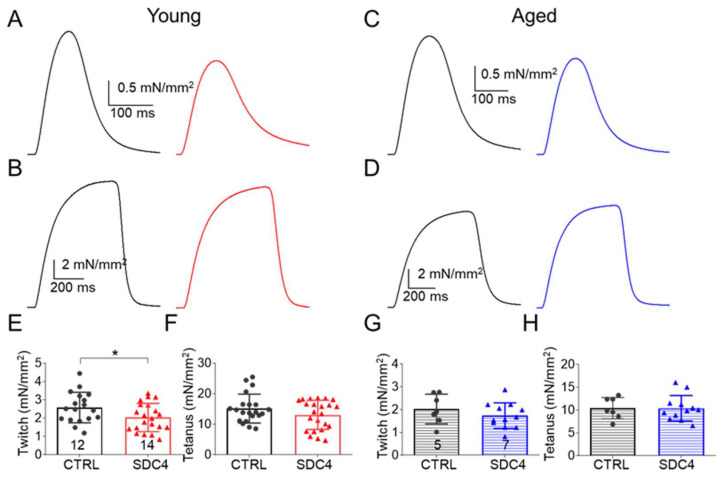
Representative ex vivo twitch (**A**,**C**) and tetanic (**B**,**D**) force on SOL muscle from young (**A**,**B**) and aged (**C**,**D**) CTRL (black) and SDC4 (red, blue) mice stimulated at 1 or 200 Hz, respectively, at room temperature (24 °C). Average twitch (**E**,**G**) and tetanic (**F**,**H**) force normalized to the cross-sectional area of the muscle. The numbers in the columns indicate the number of animals studied. * shows a significant difference from the age-matched CTRL at *p* < 0.05.

To explore the origin of the decreased in vivo muscle performance, in vitro force was studied in detail in the fast (glycolytic) *m. extensor digitorum longus* (EDL) and the slow (oxidative) *m. soleus* (SOL) muscle. Representative twitch and tetanic force in EDL (Figure 1A–D) show a decrease in the peak force in SDC4 animals. However, this decrease was present only in twitch force measured in SOL (Figure 2A–D). The decrease was statistically significant only in the twitch amplitude of both young muscle types and in aged EDL (Table 3 and Table 4), and the tetanic force showed a similar reduced tendency (Table 3 and Table 4).

**Figure 3:**
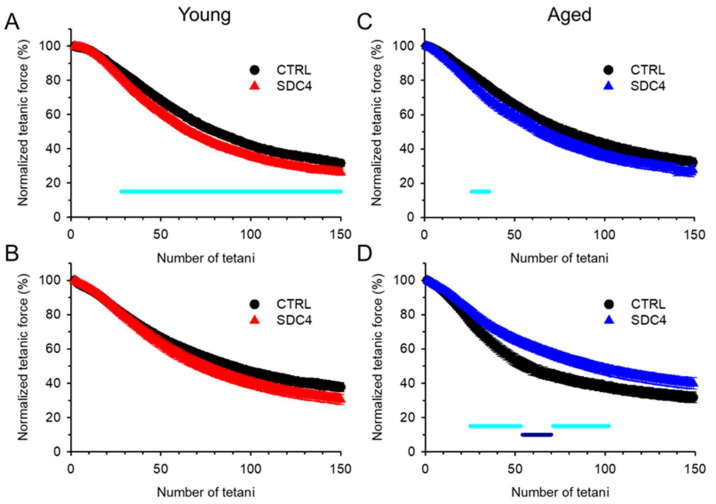
Ex vivo fatigue of EDL (**A**,**C**) and SOL (**B**,**D**) muscles from young (**A**,**B**) and aged (**C**,**D**) CTRL (black circle) and SDC4 (red and blue triangle) mice. The protocol contained 150 consecutive tetani. All tetani were normalized to the first one. The numbers of animals and muscles are the same as in Figure 1 and Figure 2. The solid light-blue and dark-blue lines show the intervals where the difference is significant between the CTRL and SDC4 mice at *p* < 0.05 and *p* < 0.01, respectively.

The cross-sectional area of the EDL and SOL muscles was identical in the groups (Table 3 and Table 4), and the time course of twitches and tetani in both muscle types was similar. We only found significant differences between the time to peak of tetanus in SOL and half-relaxation time of twitch in SOL and that of tetanus in EDL (Table 3 and Table 4). The fatigability of both muscles was also investigated with a train of 150 tetani. We observed a significant difference in fatigue in young EDL muscles (Figure 3A). This muscle showed faster fatigue in the case of the SDC4 mice than in their CTRL littermates. Furthermore, fatigue was significantly higher from the 28th tetanus onwards (Figure 3A, Table 3). The increased fatigue was present but was not significant in the case of young SOL muscle (Figure 3B, Table 4). An increased fatigability was also observed in aged EDL from the SDC4 mice (Figure 3C, Table 3), and we also found a slowed fatigue in aged SOL from animals with a decreased Syndecan-4 expression (Figure 3D, Table 4).

#### 2.2.2. Suppressed SR Ca^2+^ Release with Unaltered Voltage Dependence in SDC4 Mice

**Figure 4:**
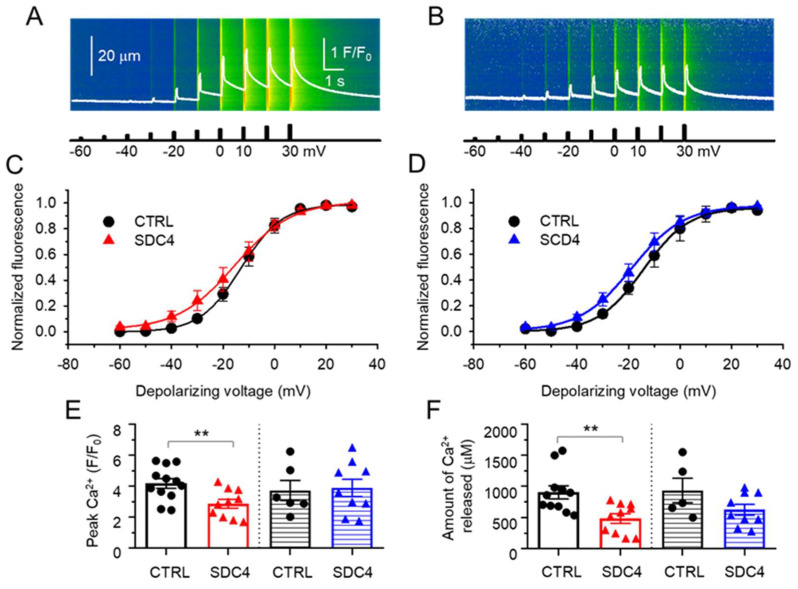
Representative line-scan images of rhod-2 fluorescence normalized to the baseline value F_0_(x) in a CTRL (**A**) and SDC4 (**B**) FDB cell from young mice subjected to successive rectangular depolarizing voltage steps under a whole-cell voltage clamp. The Ca^2+^ transients were elicited by 100 ms long progressively increasing membrane depolarizations ranging from −60 mV to +30 mV, with 10 mV increments every 1 s. The white trace is the temporal profile of the normalized fluorescence obtained by averaging 50 lines in the spatial domain normalized to average resting F_0_(x) values. Voltage dependence of the changes in [Ca^2+^]_i_ in young (**C**) and in aged (**D**) mice. The normalized F/F_0_ values were fitted with a Boltzmann function (Equation (1) in the Methods section) and then normalized to the obtained maximum for a given fibre, and, lastly, averaged over the fibres in each group. The continuous lines represent the best fit of the Boltzmann function to the average values with the following parameters: V_50_ = −13.04 and −13.66 mV, k = 7.91 and 11.23 for four young CTRL and four SDC4 mice, respectively, and V_50_ = −13.24 and −18.77 mV, k = 8.80 and 10.02 mV for four aged CTRL and five SDC4 mice, respectively. Pooled data for peak F/F_0_ values (**E**) and amount of Ca^2+^ released (**F**) obtained from single maximal depolarizing pulses (+30 mV). Empty columns represent data from young (12 fibres from 3 male and 1 female CTRL mice and 12 fibres from 3 male and 2 female SDC4 mice), dotted columns show data from aged (10 fibres from 4 male CTRL mice and 6 fibres from 5 male SDC4 mice) animals, respectively. ** shows a significant difference from the age-matched CTRL group at *p* < 0.01.

Ruling out that the altered coupling between the DHPR and RyR1 could be behind the decreased force production in SDC4 mice, calcium transients evoked by 100 ms long depolarizations covering the voltage range from −60 mV to +30 mV, with 10 mV increments were recorded in single FDBs using the whole-cell voltage-clamp technique combined with confocal microscopy. Figure 4A,B show two representative line-scan images of a CTRL and an SDC4 fibre from young animals.

The voltage dependence of the normalized fluorescence obtained from experiments similar to those shown in Figure 4A,B are shown in Figure 4C,D for FDB fibres from young and aged mice, respectively. In both cases, the activation of Ca^2+^ release from the SR in SDC4 fibres was found slightly slower, as the difference in the slope factor of release activation (k) was 3.32 and 1.22 mV in young and aged animals, respectively. However, these differences were smaller in the averaged values of k (Table 5). The calculated maximal intracellular calcium concentration ([Ca^2+^]_i,max_) showed significant differences only in the case of young animals (Table 5). These results indicate that the SDC4 downregulation does not alter the release channel activation, and the ECC machinery remains unaltered and fully operational in the SDC4 mutant. Nevertheless, the mean value of the peak of the Ca^2+^ transients obtained from single depolarization to +30 mV was smaller by 31% in the SDC4 fibres from young animals (Figure 4E); similarly, a significant decrease was not observed in the case of aged mice (Figure 4E).

The amount of Ca^2+^ released from the SR was derived from the intracellular calcium concentration ([Ca^2+^]_i_) with a simplified removal method [20,21]. Decreased Ca^2+^ release was found in FDB fibres from both young and aged SDC4 mice compared to CTRL animals (Figure 4F). However, this decrease was statistically significant only in the case of young animals.

#### 2.2.3. Altered Calcium Homeostasis in Syndecan-4-Silenced C2C12 Cells

The effect of Syndecan-4 downregulation on calcium homeostasis was also examined in a mouse skeletal muscle cell line. Endogenous expression of this regulatory protein was demonstrated earlier in C2C12 myotubes with decreased Syndecan-4 levels during differentiation [22]. Here, we used clones with a stable downregulation of Syndecan-4 showing a decreased proliferation capacity [9] to examine calcium homeostasis at rest, following depolarization, and during migration.

**Figure 5:**
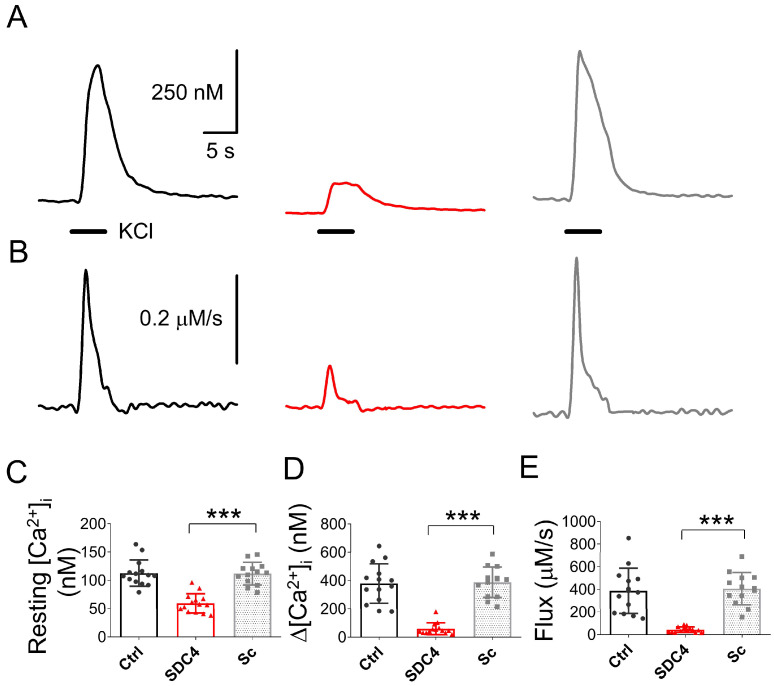
Representative KCl depolarization-evoked Ca^2+^ transients measured on 5-day-old differentiated control (Ctrl, black), Syndecan-4-downregulated (KD, red), and scrambled (Sc, grey) C2C12 myotubes (**A**). (**B**) Calcium release from the SR, calculated from the traces in panel A. (**C**) Pooled data for resting [Ca^2+^]_i_. (**D**) The maximal increase in [Ca^2+^]_i_ upon KCl depolarization. (**E**) Peak calcium flux release from the SR. The protocol of the solution exchange in panel A was 5 s long 120mM KCl in normal Tyrode’s solution (black horizontal solid lines also show the level of zero). The number of cells averaged was 14, 14, and 13 from 3 independent control (Ctrl), Syndecan-4-KD (KD), and scrambled (Sc) cell cultures, respectively. *** denotes a significant difference from the scrambled group at *p* < 0.001.

5-day-old control (nontransfected), shRNA-mediated Syndecan-4-knockdown (KD), and scrambled (Sc) C2C12 myotubes were used for functional experiments. The resting intracellular calcium concentration was similar (*p* > 0.2) in both control and scrambled groups, while it decreased significantly in Syndecan-4-KD cells (Figure 5C).

The calcium transients were elicited by depolarization using 120 mM KCl in normal extracellular calcium concentration (1.8 mM). Figure 5A presents representative calcium transients in control, in Syndecan-4 KD, and scrambled myotubes. The corresponding Ca^2+^ release from the SR, calculated from traces in Figure 5A, is shown in Figure 5B. Similarly to the resting [Ca^2+^]_i_ (Figure 5C), the amplitude of the depolarization-evoked calcium transients that were almost identical to the control and scrambled cells (Figure 5D). In contrast, the amplitudes of the KCl-evoked calcium transients in Syndecan-4 KD cells were significantly reduced (Figure 5D). Similar results were obtained for the Ca^2+^ release flux, which was significantly reduced in Syndecan-4 KD cells, and almost identical in the control and scrambled myoblasts (Figure 5E).

Syndecan-4 KD myoblasts were shown to have reduced migration capability compared to control C2C12 cells [9]. The measurement of whole-cell [Ca^2+^]_i_ in migrating cells gives the possibility of studying the functional properties of calcium homeostasis during motility. Thus, intracellular calcium concentration was measured during migration assays. Supplementary Figure 1A shows representative images 4 h after removing the silicone insert from the scrambled cell culture dish, allowing the cells to migrate into the cell-free zone.

**Figure 6:**
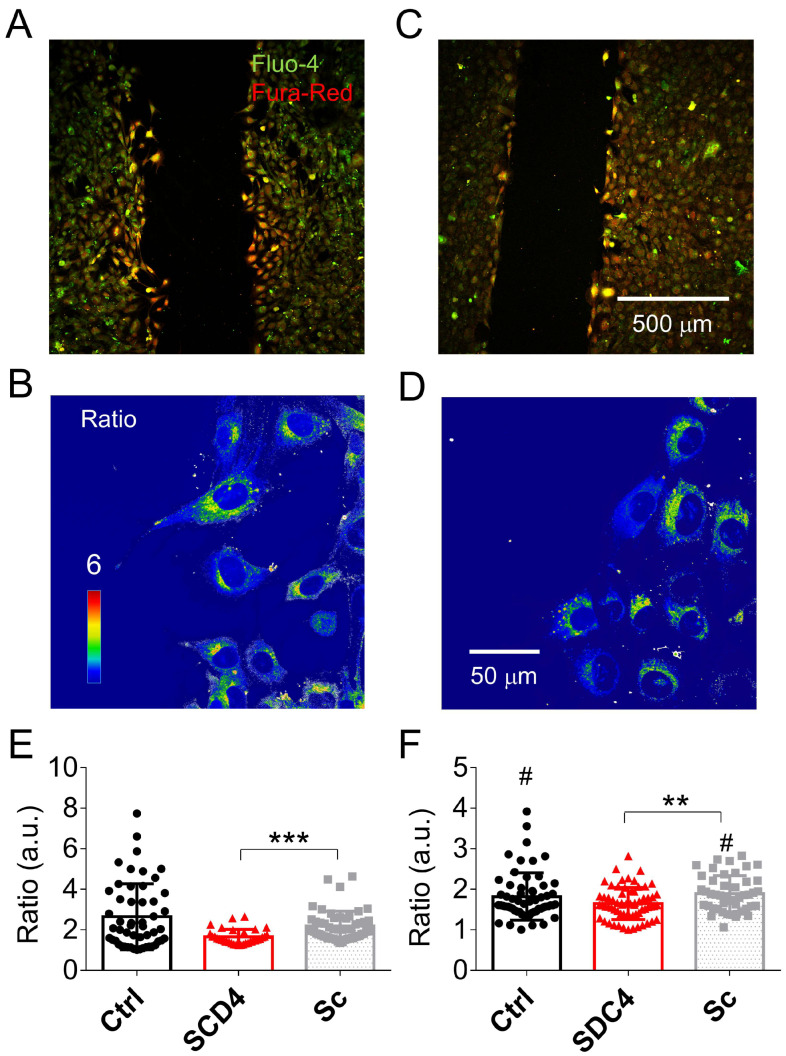
Representative low magnification merged images 4 h after removing the insert from the scrambled (**A**) and Syndecan-4 KD (**C**) cell culture. High magnification ratio image showing migrating scrambled (**B**) and Syndecan-4 KD (**D**) cells. The average fluorescent ratio of the migrating (**E**) and nonmigrating (**F**) control (black), knockdown (red), and scrambled (grey) cells. The number of cells averaged was 52, 33, and 26 in migrating and 61, 65, and 46 in nonmigrating from 3 independent control (Ctrl), Syndecan-4 KD (KD), and scrambled (Sc) cell cultures, respectively. **, and *** denote significant difference from scrambled cell cultures at *p* < 0.01, and *p* < 0.001, respectively. # denotes a significant difference from migrating cells from the same group at *p* < 0.05.

As demonstrated, a number of cells started to migrate, and cells in the migration zone showed higher fluorescent intensities than the nonmigrating cells at both excitation wavelengths (Supplementary Figure S1A,B). A similar observation was made in scrambled and Syndecan-4 KD cells (Figure 6A–D). Nevertheless, control and scrambled cells presented higher migration capacities than Syndecan-4 KD cells. The analysis of the fluorescent ratios revealed that migrating cells had higher [Ca^2+^]_i_ compared to nonmigrating cells in the control and scrambled cell cultures (Figure 6E,F). However, we did not find a significant difference in [Ca^2+^]_i_ between Syndecan-4 KD cells in the migration or in the nonmigration zones (Figure 6E,F). Moreover, KD cells showed significantly lower fluorescent ratios—i.e., lower [Ca^2+^]_i_—compared to scrambled cells, independent of their migration capacity.

## 3. Discussion

In this study, we have characterized the in vivo and in vitro muscle force and analyzed the calcium homeostasis in Syndecan-4 (SDC4)-deficient mice and murine C2C12 skeletal muscle cells. When compared to WT, we found that SDC4 mice had reduced in vivo and in vitro muscle force and decreased calcium release from the SR with unaltered voltage dependence of the calcium transients elicited by membrane depolarization. Intracellular calcium handling after depolarization and during migration of 5-day-old C2C12 myotubes was also significantly altered following SDC4 silencing.

Currently, no data are available regarding the effects of Syndecan-4 downregulation or complete loss in aged (older than 20 months) animals, thus, we decided to perform experiments on aged Syndecan-4^+/–^ (SDC4) mice. We did not observe any difference in viability and weight gain between the SDC4 and CTRL mice. The slight 7% reduction in body weight found in young SDC4 animals compared to CTRL animals was similar in aged mice (Table 1) as well. Interestingly, the reduced weight of EDL (92%) and SOL (89%) muscles observed in young SDC4 mice was reversed in aged animals (120% and 123% for EDL and SOL, respectively) (Table 3 and Table 4). This perhaps implies long-term muscle remodelling, which could potentially be a compensatory mechanism in SDC4 mice.

The reduced muscle performance we observed here may be partly due to the decreased muscle weight and increased number of muscle fibres with a smaller cross-sectional area, a phenomenon described earlier by Ronning et al. [16] in *m. tibialis anterior* muscle of 10-week-old animals. However, we did not investigate the diameters of single fibres, and assumed similar changes, since we also found smaller muscle weights of EDL (Table 3) and SOL (Table 4) of young SDC4 mice compared to CTRL animals. This decrease in muscle weight converted into an increase in aged SDC4 animals, and this was accompanied by the increased cross-sectional area of the muscles (Table 3 and Table 4). Earlier, we showed that Syndecan-4 alters the organization of the actin cytoskeleton in C2C12 cells. dSTORM super-resolution microscopy results have revealed that Syndecan-4 affects the nanoscale structure of the actin network in the lamellipodia of C2C12 myoblasts during migration [9] and also in differentiating cell cultures [14]**,** thereby influencing cell migration and cell–cell fusion. Since Syndecan-4 regulates the activity of small GTP-ase Rac1 [22], the Syndecan-4/Rac1-mediated actin remodelling [14] plays a role in this phenomenon. Moreover, Syndecan-4 directly binds alpha-actinin, a cross-linking protein between F-actin filaments [23]. These roles of Syndecan-4 in actin organization raise the possibility of its participation in the organization of contractile proteins, which might explain the observed changes in muscle performance in the lack of Syndecan-4. Altogether, the fact that the decrease in muscle force remained in aged animals on a similar level as it was in young SDC4 mice (Figure 1 and Figure 2, Table 3 and Table 4) supports the idea that Syndecan-4 has a crucial role during myogenesis and becomes less important if the muscle is already developed.

During the in vitro force measurement, we only found significantly reduced force generation in the case of single twitches in the EDL and SOL of SDC4 mice. For tetani, we saw a similar tendency as for the twitches, i.e., they were also smaller when compared to the CTRL, but this change was not statistically significant in both types of examined muscles regardless of age (Figure 1 and Figure 2, Table 3 and Table 4). Performing a closer inspection of the timing of force transients, we found that the activation and relaxation of contraction were on average unchanged. We encountered only a significantly slower half-relaxation time of tetani in young and faster time to peak and half-relaxation (thus duration) of twitch in aged EDL muscles (Table 3). Similarly, a faster half-relaxation time of twitches and a slower time to peak of tetani was observed in young, and a faster time to peak and half relaxation (thus duration) of twitch and tetani in aged SOL muscles (Table 4). The impaired activation and relaxation could also be the result of the modified elasticity of muscle fibres. It was proposed that Syndecan-4 can facilitate the effects of the extracellular matrix on convergent extension movements [24]. All of these findings may explain the reduced muscle work of SDC4 muscles, which is more pronounced in aged specimens when compared to similarly aged wild-type muscles. The fatigability of both muscles investigated increased, but this only reached a significant level in the case of young EDL at the end of the fatigue protocol (Figure 3, Table 3 and Table 4). For both examined muscle types from aged animals, the fatigue was significantly faster, but altogether, its final level was identical in SDC4 and CTRL mice (Figure 3, Table 3 and Table 4). This data suggests that Syndecan-4 can exert more pronounced effects in glycolytic muscle types (EDL) than in oxidative (SOL) ones (in young animals), and this difference disappears in aged animals. The mild effects on skeletal muscle force production suggest that some compensatory mechanism should exist which helps to maintain the force generation on an expected physiological level. It was shown earlier that when Syndecan-4 was missing from the muscle, Syndecan-2 expression increased almost four times [16]. Moreover, the increased expression of Syndecan-2 has also been found as a compensatory mechanism during cartilage growth [25]. This, however, failed to be validated in the heart tissue of Syndecan-4 knockout mice [26]. Earlier in C2C12 cells, we successfully verified the compensatory upregulation of Syndecan-1,-2, and -3 expression upon Syndecan-4 KD [22]. Based on the above, one can suggest that the replacement of Syndecan-4 by Syndecan-2 may be tissue-specific.

**Figure 7:**
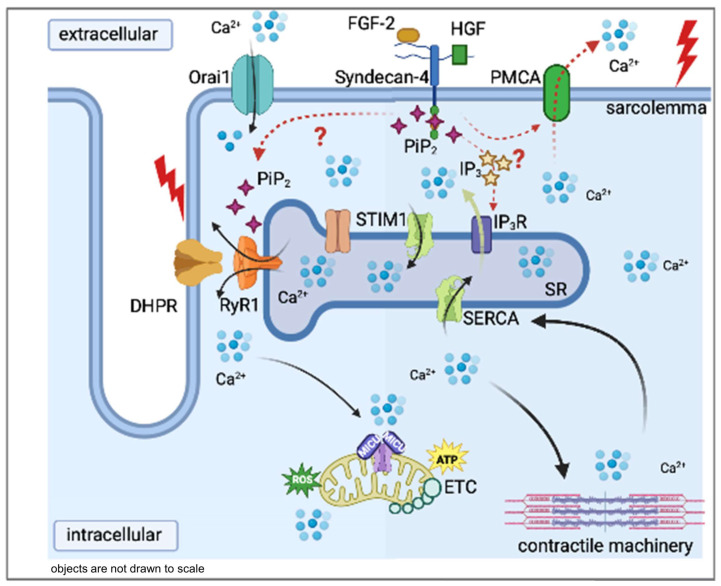
Proposed interactions of Syndecan-4 in skeletal muscle. Syndecan-4 may directly alter the plasma membrane calcium pump (PMCA). It may indirectly modify the opening of type 1 ryanodine receptor (RyR1) in the sarcoplasmic reticulum (SR) membrane by modifying the PiP_2_ concentration in the intracellular space. The black arrows show the path of calcium ions in the intracellular space. Red arrows tackle the connection between the proposed interactions. The green arrow tackles the Ca^2+^ efflux from the SR via IP_3_ receptors, a phenomenon seen only in skeletal myotubes and not in adult muscle fibres. Further abbreviations: DHPR: dihydropyridine receptor; ETC: electron transport chain; FGF-2: fibroblast growth factor type-2; HGF: hepatocyte growth factor; IP_3_: inositol 1,4,5-trisphospate; IP_3_R: inositol 1,4,5-trisphospate receptor; MICU: mitochondrial calcium uniporter complex; Orai1: calcium release-activated channel 1; SERCA: Sarco(endo)plasmic reticulum calcium pump; STIM1: stromal interaction molecule; IP3R: inositol trisphosphate receptor.

Previous experiments on cell cultures have shown that Syndecan-4 can modify the intracellular Ca^2+^ concentration in different cell types. Our current findings showing the reduced resting intracellular Ca^2+^ concentration and overall altered calcium handling during migration in Syndecan-4 knockdown (KD) C2C12 cells are illustrated in Figure 5C. The literature tackling the effects of the lack of Syndecan-4 in different cell types is rather conflicting. On one hand, the TRPC6-mediated calcium influx was found to be reduced in Syndecan-4 knockdown podocytes by decreasing the number of channel proteins [7]. On the other hand, in another study using rat embryonic fibroblasts, the reduction in Syndecan-4 led to elevated cytosolic calcium concentrations [8]. This probably arose from more TRPC7 calcium channels being open in the absence of Syndecan-4. Lastly, a recent study by Becski et al. [9] also showed decreased resting intracellular Ca^2+^ concentrations in migrating Syndecan-4 KD C2C12 cells. These data are consistent with our present results obtained on migrating C2C12 myotubes kept in culture for 5 days (Figure 6), and may have several explanations: (i) Increased SERCA pump activity can significantly reduce the [Ca^2+^]_i_ by pumping more calcium back into the SR. However, at the moment, there is no experimental evidence for any connection between the Syndecan-4 expression and the SERCA activity. (ii) Increased plasma membrane calcium pump (PMCA) activity can also significantly reduce the [Ca^2+^]_i_ by pumping out more calcium from the cytosol. This possibility is easier to imagine since Syndecan-4 is also located in the plasma membrane, and can thus modulate the pump activity directly. Nevertheless, we have to admit that neither the former nor the latter pump mechanisms are on the list of possible interaction partners of syndecans published by Gondelaud and Ricard-Blum in their in-depth review on syndecans [27]. Furthermore, DHPR and RyR1 are also not included in this list of putative interaction partners, and we have shown here that the voltage dependence of calcium transients did not change in FDB fibres from SDC4 mice. On the other hand, phosphatidylinositol 4,5-bisphosphate (PiP_2_) is on the list as a partner with a direct connection point on the core of Syndecan-4 [28]. Notably, the phosphorylation of the cytoplasmic domain of Syndecan-4 affects PiP_2_ binding [28]. In previous work, we have demonstrated that PiP_2_ has a great impact on [Ca^2+^]_i_ in skeletal muscle [29]. In certain pathological conditions, such as myotubular myopathy (MTM1), elevated levels of PiPs can decrease the intracellular Ca^2+^ concentration leading to weak muscles. Based on this, we hypothesize that if the lack of Syndecan-4 may lead to the increase in free PiP_2_ (or at least the local increase in specific subcellular regions), this could explain the decreased twitch force we observed in SDC4 mice (Figure 7). Furthermore, PiP_2_ may have a role in the development of slow intracellular calcium waves in myotubes via IP_3_ pathways [30]. In differentiating skeletal muscle myotubes, RyRs and IP_3_ receptors are present simultaneously in the membrane of the SR. While the former is responsible for the calcium release and serves the contraction, the latter could supply the rise in nucleoplasmic calcium, which can be involved in gene transcription. This, and the aforementioned double role of PiP_2_, may explain the dramatic decrease in the [Ca^2+^]_i_ that we found in the SDC4 KD myotubes (Figure 5). If the maturation of skeletal muscle is altered in the lack of Syndecan-4, the modified gene expression could lead to a smaller muscle size that we observed in young SDC4 mice (Table 3) and a smaller fibre size found previously [16].

The absence of normal Syndecan-4 can modify the metabolism of the body. It was shown in drosophila carrying mutated Syndecan-4 that the mitochondrial respiration and the storage of fat were reduced [31,32]. Furthermore, similar effects were observed in humans in the presence of single nucleotide polymorphisms in the human SDC4 gene. Patients carrying this mutation had lower levels of glucose and higher resting energy expenditure. They were more sensitive to environmental stress and had reduced metabolic activity in normal diet conditions [31]. We can assume similar changes in the metabolism (i.e., glucose level) of the skeletal muscle of SDC4 mice, which can also explain the reduced physical activity of these animals. However, to clarify this possibility, further experiments have to be carried out.

In conclusion, our current results indicate that processes involved in maintaining adequate intracellular calcium levels and force development in skeletal muscle were somewhat affected by Syndecan-4 deficiency in young and aged mice, as well as in differentiating C2C12 myotubes. The aforementioned changes were observed both in vivo and in vitro. To better understand the molecular mechanism underlying these changes, further targeted studies are needed, which may help to elucidate the exact role of Syndecan-4 in skeletal muscle in health and disease. These new findings may have significant importance for elucidating the mechanism of the regeneration of injured muscles in both young and aged individuals.

## 4. Materials and Methods

### 4.1. Animal Care and Genotyping

All animal experiments followed the guidelines of the European Community (86/609/EEC). The experimental protocol was approved by the Institutional Animal Care Committee of the University of Debrecen (3–1/2019/DEMAB). The mice were kept in plastic cages with metal grid covers and fed with pelleted mouse chow and tap water ad libitum. Room lighting was an automated cycle of 12 h light starting at 6 am and ending at 6 pm. The room temperature was kept within the range of 22–25 °C. The Syndecan-4 knockout mouse strain was kindly provided by Dr James R. Whiteford (William Harvey Research Institute, Queen Mary University of London, London, England). The generation of Syndecan-4 knockout mice has been described by Ishiguro et al. [33]. The mouse colony was maintained by intercrossing Syndecan-4 heterozygous mice, and the genotype of the progeny was determined by polymerase chain reaction, as described in the Supplementary Materials. Representative genotyping results are shown in Supplementary Figure S2. Experiments were performed using control (CTRL) and Syndecan-4 knockdown (SDC4) (heterozygous) mixed gender, young (2–5 months old weighing 20–30 g) and aged (18–24 months old, weighing 26–36 g) mice.

### 4.2. In Vivo Experiments

#### 4.2.1. Voluntary Activity Wheel Measurement

Male mice from both groups were singly housed in a cage equipped with a mouse running wheel (Campden Instruments Ltd., Loughborough, UK). The wheels were connected to a computer, and rotations were continuously recorded at 20 min intervals for 14 days. The daily average and the maximal speed, the distance covered, and the duration of running were calculated for each mouse and then averaged by groups.

#### 4.2.2. Forepaw Grip Test

The force of the forelimbs was measured, as described earlier [34]. Briefly, the mouse was placed over the grid horizontally and allowed only its forepaws to grasp the grid. Then, the mouse was gently pulled back by its tail and the maximal force when the animal released the grid was digitized and stored by an online-connected computer. The test was repeated at least 10 times and averaged to obtain a single data point. For all animal groups, the grip test was performed on the day when the mouse was sacrificed.

### 4.3. In Vitro Experiments

Animals were anaesthetized and sacrificed using the authorized protocol outlined by the Animal Care Committee of the University of Debrecen (3-1/2019/DE MAB). Following anaesthesia with isoflurane (5%) and cervical dislocation, the *m. extensor digitorum longus* (EDL), the *m. flexor digitorum brevis* (FDB), and *m. soleus* (SOL) from the hind limb were dissected.

#### 4.3.1. Measurement of Muscle Force

Muscle contractions were measured, as described previously [34]. In brief, fast (EDL) and slow (SOL) muscles were mounted horizontally in an experimental chamber continuously superfused (10 mL/min) with Krebs’ solution (containing 135 mM NaCl, 5 mM KCl, 2.5 mM CaCl_2_, 1 mM MgSO_4_, 10 mM HEPES, 10 mM glucose, and 10 mM NaHCO_3_; pH 7.2; room temperature) equilibrated with 95% O_2_ plus 5% CO_2_. One end of the muscle was fixed with a pin via the tendon, while the other was fixed to a capacitive mechano-electric force transducer (Experimetria, Budapest, Hungary). Two platinum electrodes placed below the muscle were used to stimulate the muscles. Single twitches were elicited with short (2 ms) supramaximal pulses. Force responses were digitized at 2 kHz using a Digidata 1200 A/D card and stored using Axotape software (Axon Instruments, Foster City, CA, USA) on an online-connected computer. Muscle length was then adjusted by positioning the transducer to produce the maximal force response and allowing equilibration for 5 min.

Single twitches were elicited with 2 ms long pulses at 0.5 Hz. At least 10 consecutive twitches were measured under these conditions from every muscle. If the amplitude of the force transients within such a train varied by less than 3%, the average amplitude of all transients was used to describe the given muscle. Otherwise, the measurement was interrupted, and the muscle was excluded from the experiment. Tetanus was elicited using a train of single pulses with a frequency of 200 Hz for 200 ms (EDL) or 100 Hz for 500 ms (SOL). The duration of a twitch or tetanus was determined by computing the time between the onset of the transient and the relaxation to 10% of maximal force.

The fatigue protocol comprised 150 tetanic stimulations at 200 Hz with a 200 ms duration for EDL and at 100 Hz with a 500 ms duration for SOL, every 2 sec. The fatigue of muscles was determined by normalizing all tetani in the series to the first one.

#### 4.3.2. Isolation of Single Skeletal Muscle Fibres

Single skeletal muscle fibres from the FDB muscle of the mouse were used in all calcium concentration measurements. Calcium-free Tyrode’s solution (containing 137 mM NaCl, 5.4 mM KCl, 0.5 mM MgCl_2_, 5 mM EGTA, 11.8 mM HEPES, and 1 g/L glucose; pH 7.4) was used during the dissection of the muscle. Individual muscle fibres from FDB were enzymatically dissociated in minimal essential media containing 0.2% Type I collagenase (Sigma-Aldrich, St. Louis, MO, USA) at 37 °C for 65 min [35,36]. The FDB muscles were triturated gently to release single fibres in normal Tyrode’s solution (similar to the above except: 1.8 mM CaCl_2_ and 0 mM EGTA). The isolated fibres were then stored in culture dishes at 4 °C in the fridge for further use.

#### 4.3.3. Voltage Clamp and Calculation of Intracellular Ca^2+^ Concentration

The experimental protocol was described earlier [37]. Briefly, isolated single FDB fibres were voltage-clamped (Axoclamp 2B, Axon Instruments) and imaged using a confocal microscope (Zeiss 5 Live, Oberkochen, Germany). Fibres were dialyzed with an internal solution containing rhod-2 through a glass patch pipette. Experiments were carried out at room temperature (20–22 °C) and the holding potential was set to −80 mV. The pipette resistance varied between 1 and 2 MΩ. The measurements were performed in the presence of 10 mM EGTA so that the endogenous calcium buffers in the removal process were nearly negligible. Correction for linear capacitive currents was achieved using analogue compensation.

The external bath solution: 140 mM TEA-CH3SO3, 1 mM CaCl_2_, 3.5 mM MgCl_2_, 10 mM HEPES, 1 mM 4-AP, 0.5 mM CdCl_2_, 0.3 mM LaCl_3_, 0.001 mM TTX (citrate), and 0.05 mM BTS (N-benzyl-p-toluene sulphonamide). The pH was adjusted to 7.2 using TEA-OH, and the osmolality was adjusted to 320 mOsm using TEA methanesulfonate.

The internal (pipette) solution: 110 mM N-methylglucamine, 110 mM L-glutamic acid, 10 mM EGTA, 10 mM Tris, 10 mM glucose, 5 mM Na–ATP, 5 mM phosphocreatine Tris, 0.1 mM rhod-2, 3.56 mM CaCl_2_, and 7.4 mM MgCl_2_ were added for a nominal 1 mM [Mg^2+^] and 100 nM [Ca^2+^]. The pH was set to 7.2 with NaOH, and the osmolality was set to 320 mOsm using N-methylglucamine.

The voltage dependence of intracellular Ca^2+^ concentration ([Ca^2+^]_i_) change was described by Boltzmann function:

[Ca^2+^]_i_(V_m_) = [Ca^2+^]_i,max_/(1+exp(−(V_m_ − V_50_)/k) (1)

to derive the transition voltage V_50_ and limiting logarithmic slope 1/k. To visualize the voltage dependence, the activation peaks of the calcium transients were normalized to [Ca^2+^]_i,max_ and plotted as a function of the actual membrane potential.

#### 4.3.4. Confocal Microscopy and Image Analysis

All confocal images were recorded using a 20× air-immersion objective (NA: 1.0) in an inverted confocal microscope. The calcium-sensitive dye, rhod-2, was excited at 543 nm, and the emitted light above 550 nm was collected using a long-pass filter. In the patch clamp studies, the line-scan confocal image acquisition was synchronized using pClamp 11.0 software (Molecular Devices, San Jose, CA, USA) with the application of 100 ms long single or repetitive depolarizations. The frequency of scanning a line was 2 kHz, and the spatial resolution was 0.24 μm/line. Voltage-evoked Ca^2+^ transients were analysed using in-house software, taking into account the dissociation constant for rhod-2 (K_d_(rhod-2) = 1.58 µM) and k_ON_ = 0.07 µM^−1^ ms^−1^ [38]. The baseline fluorescence (F_0_[x]) was calculated by averaging between 15 and 20 lines in the time domain before the first depolarizing pulse. The fluorescence intensity was presented after normalization to F_0_[x] (F[x]/F_0_[x]).

#### 4.3.5. Cell Culture and Plasmids

For in vitro experiments, C2C12 mouse myoblast cells (ATCC; Manassas, VA, USA) were cultured in Dulbecco’s modified Eagle’s medium with high glucose content (4.5 g/L glucose containing 584 mg/L glutamine and 110 mg/L pyruvate; Corning, NY, USA) supplemented with 20% foetal bovine serum (Gibco/Thermo Fisher Scientific, Waltham, MA, USA) and 50 µg/mL gentamicin (Lonza, Basel, Switzerland). The downregulation of Syndecan-4 was described earlier [9]. In brief, to achieve Syndecan-4 silencing, C2C12 cells were stably transfected with plasmids expressing short hairpin RNAs (shRNAs) specific to mouse Syndecan-4 (KD cells) or a scrambled target sequence. The plasmids were obtained from OriGene (TR513122; Rockville, MD, USA) and targeted the following sequences: 5′-GAA CTG GAA GAG AAT GAG GTC ATT CCT AA-3′ (specific for Syndecan-4), and 5′-GCA CTA CCA GAG CTA ACT CAG ATA GTA CT-3′ (scrambled). For transfection, an X-tremeGENE transfection reagent (Roche, Basel, Switzerland) was used according to the manufacturer’s recommendation. Then, transfected cells were selected in a puromycin-containing medium (4 µg/mL). To carry out further studies, cells were seeded on glass coverslips for 24 h, and differentiation was then induced by replacing the growth medium with a differentiation medium containing 2% horse serum (Gibco/Life Technologies, New Zealand). The Syndecan-4 expression of normal and mutated C2C12 cells was compared using Western blot and qPCR experiments [9,14,22]. The silencing of Syndecan-4 decreased its level by ~50% in the KD cells.

#### 4.3.6. Whole-Cell Calcium Measurements in Cell Culture

Changes in [Ca^2+^]_i_ were monitored using the calcium-sensitive fluorescent dye Fura 2, as reported earlier [36]. C2C12 myotubes were loaded with Fura 2 AM (10 µM) for 1 h (37 °C, 5% CO_2_) in DMEM supplemented with 10% FBS and neostigmine to inhibit choline-esterases. The coverslip containing the myotubes was then placed on the stage of an inverted fluorescent microscope (Nikon Diapoth, Tokyo, Japan). Measurements were performed in normal Tyrode’s solution. In the depolarizing solution, 120 mM NaCl was exchanged for KCl. During the measurement, the excitation wavelength was alternated at 50 Hz between 340 and 380 nm using a dual-wavelength monochromator (Deltascan, Photon Technology International, New Brunswick, NJ, USA), while the emitted light was monitored at 510 nm using a photomultiplier at 10 Hz. [Ca^2+^]_i_ was calculated from the ratio of fluorescence intensities (R = F_340/F380_) using an in vivo calibration (R_min_ = 0.20, R_max_ = 19.87, and K_d_•β = 943.37). Resting [Ca^2+^]_i_ was calculated by averaging the ratio before the application of 120 mM KCl depolarization in normal Tyrode’s solution. The Ca^2+^ flux from the SR into the cytoplasm was calculated using a model that took into account the Ca^2+^ binding to intracellular binding sites and the removal of Ca^2+^ from the intracellular space back into the SR, as described earlier [39].

#### 4.3.7. Calcium Concentration Measurements during Migration

The migration assay of C2C12 cells was performed following Becsky et al. [9]. Briefly, cells (2 × 104) were plated on a glass coverslip with a 500 µm wide silicon insert (Ibidi GmbH, Gräfelfing, Germany) and allowed to adhere to the surface for 24 h. Before the removal of the insert, Mitomycin C (Sigma) treatment was applied (10 µg/µL, 2 h) to block cell proliferation. PBS was then applied to wash out the Mitomycin C. C2C12 myoblasts were kept in a solution (125 mM K-glutamate, 10 mM HEPES, 1 mM EGTA, 5 mM MgCl_2_, 5 mM Na–ATP, 10 mM Na–phosphocreatine, 10 mM glucose, 0.13 mM CaCl_2_, and 8% dextran) containing 2 mM Fluo-4, AM, and 1 mM Fura Red, AM, for 1 h at 37 °C. This solution was then exchanged for a dye-free extracellular solution. The [Ca^2+^]_i_ during migration was monitored at different time points at 22 °C using an LSM 510 LIVE confocal laser scanning microscope (Zeiss, Oberkochen, Germany).

Calcium imaging was performed with two-dimensional scanning using a 40× oil-immersion objective (NA: 1.3). The fluorescence intensity was measured using a 488 nm excitation wavelength and two detection channels: for Fluo-4 a bandpass (500–525 nm), while for Fura Red, a long-pass (>635 nm) filtered channel. There was no crosstalk between the two channels due to the wide gap between the cut-off wavelengths. The fluorescence intensity of the two dyes was stored parallel in two separate images. Image pairs were analyzed manually using the ImageJ program (National Institutes of Health, Bethesda, MD, USA, https://imagej.nih.gov/ij/ accessed on 30 July 2021.). First, the average background fluorescence was calculated by manually selecting a cell-free area of the image, and this value was subtracted from the average fluorescence of the cells. The area of cells was also selected manually and was identical in both images. The average fluorescence of Fluo-4 and Fura Red on the cells was calculated in ImageJ, and their ratio was taken as a value that corresponds to the actual [Ca^2+^]_i_.

#### 4.3.8. Chemicals and Statistical Analysis

Chemicals, unless otherwise stated, were purchased from Sigma-Aldrich (St. Louis, MO, USA) and were of analytical grade.

Pooled data were expressed as mean ± standard error of the mean (SEM). All data sets passed the Kolmogorov–Smirnov normality test. The differences between control and SDC4 animals or control, scrambled, and Syndecan-4 knockdown C2C12 cells were assessed using one-way analysis of variance (ANOVA) and the pairwise Bonferroni’s multiple comparison method using the Prism statistical program (GraphPad Software, San Diego, CA, USA). F-tests were used to test significance, and a *p*-value of less than 0.05 was considered statistically significant.

## Figures and Tables

**Table 1:** Forepaw grip force measurement.

	Young		Aged	
	CTRL	SDC4	CTRL	SDC4
Body weight (g)	26.7 ± 1.1	24.9 ± 0.9	32.8 ± 0.8	30.1 ± 0.5 **
Maximal force (mN)	148.8 ± 7.2	126.1 ± 3.8 **	154.3 ± 7.3	134.1 ± 4.5 *
Force normalized to body weight (mN/g)	55.8 ± 1.5	51.1 ± 1.2 *	50.9 ± 2.3	43.8 ± 2.0 *
Number of mice	9	12	11	18
Gender distribution	4 male, 5 female	8 male, 4 female	11 male	18 male

* and ** show significant differences from the CTRL in the same age group at *p* < 0.05 and *p* < 0.01, respectively.

**Table 2:** Voluntary running measurement.

	Young		Aged	
	CTRL	SDC4	CTRL	SDC4
Distance (m/day)	7335.7 ± 155.6	7571.1 ± 279.6	3203.7 ± 206.8	2231.7 ± 177.0 **
Average speed (m/min)	14.3 ± 0.2	11.5 ± 0.5 ***	5.2 ± 0.3	4.2 ± 0.2 *
Max speed (m/min)	27.4 ± 0.2	23.2 ± 1.1 **	12.3 ± 0.4	9.7 ± 0.5 **
Time (min/day)	516 ± 6	631 ± 7 ***	326 ± 10	270 ± 14 **
Number of mice	4	5	4	4

*, **, and *** show significant differences from the CTRL in the same age group at *p* < 0.05, *p* < 0.01, and *p* < 0.001, respectively. All mice were male.

**Table 3:** Parameters of twitch and tetanus in EDL.

	Young				Aged			
	Twitch		Tetanus		Twitch		Tetanus	
	CTRL	SDC4	CTRL	SDC4	CTRL	SDC4	CTRL	SDC4
Number of muscles	17	18	17	18	7	12	7	12
Muscle weight (mg)	15.5 ± 0.5	14.4 ± 0.3 *	15.5 ± 0.5	14.4 ± 0.3 *	13.9 ± 0.4	17.1 ± 0.3 ***	13.9 ± 0.4	17.1 ± 0.3 ***
Peak force (mN)	2.04 ± 0.10	1.77 ± 0.08 *	10.16 ± 0.35	9.34 ± 0.31	1.74 ± 0.26	1.81 ± 0.13	7.31 ± 0.55	8.05 ± 0.31
Force (mN/mm^2^)	1.99 ± 0.15	1.65 ± 0.09 *	9.96 ± 0.72	8.79 ± 0.49	2.04 ± 0.27	1.54 ± 0.09 *	8.75 ± 0.73	6.93 ± 0.31 *
TTP (ms)	32.9 ± 1.4	29.9 ± 0.2	172.2 ± 4.0	157.7 ± 6.7	38.1 ± 3.1	30.9 ± 1.2 *	193.5 ± 4.6	192.2 ± 3.1
HRT (ms)	30.1 ± 0.8	26.6 ± 0.9	73.4 ± 4.1	99.5 ± 7.1 **	27.6 ± 1.5	22.9 ± 0.6 **	81.9 ± 7.2	79.3 ± 4.3
Duration (ms)	210.2 ± 29.1	164.4 ± 13.1	336.4 ± 5.4	338.5 ± 4.2	207.7 ± 25.2	132.0 ± 5.6 **	374.4 ± 12.9	356.5 ± 6.6
CSA (mm^2^)	1.07 ± 0.06	1.11 ± 0.05	1.07 ± 0.06	1.11 ± 0.05	0.84 ± 0.06	1.18 ± 0.07 **	0.84 ± 0.06	1.18 ± 0.07 **
Fatigue at 50 (%)			31.6 ± 1.7	38.7 ± 2.2 *			33.3 ± 1.6	40.5 ± 3.1
Fatigue at 100 (%)			57.7 ± 1.8	64.8 ± 2.0 *			57.0 ± 2.7	63.6 ± 2.9
Fatigue at 150 (%)			68.4 ± 1.9	74.1 ± 1.8 *			67.9 ± 2.6	72.2 ± 2.7

Force parameters in the case of young animals from 9 (5 male and 4 female) and 10 (6 male and 4 female), in case of aged animals from 5 (2 male and 3 female) and 7 (5 male and 2 female) control and Syndecan-4 KD mice, respectively. *, **, and *** show significant difference from the control at *p* < 0.05, *p* < 0.01, and *p* < 0.001, respectively.

**Table 4:** Parameters of twitch and tetanus in SOL.

	Young				Aged			
	Twitch		Tetanus		Twitch		Tetanus	
	CTRL	SDC4	CTRL	SDC4	CTRL	SDC4	CTRL	SDC4
Number of muscles	18	18	18	18	7	12	7	12
Muscle weight (mg)	16.3 ± 0.7	14.5 ± 0.2 **	16.3 ± 0.7	14.5 ± 0.2 **	14.0 ± 0.5	16.8 ± 0.6 **	14.0 ± 0.5	16.8 ± 0.6 **
Peak force (mN)	2.17 ± 0.12	1.76 ± 0.11 *	12.93 ± 0.30	11.33 ± 0.58 *	1.70 ± 0.15	1.77 ± 0.13	8.85 ± 0.56	10.17 ± 0.48
Force (mN/mm^2^)	2.58 ± 0.20	2.03 ± 0.17 *	15.12 ± 1.03	13.05 ± 1.00	2.03 ± 0.25	1.73 ± 0.16	10.42 ± 0.88	10.41 ± 0.81
TTP (ms)	74.1 ± 3.5	70.3 ± 2.4	507.5 ± 1.9	520.8 ± 1.9 ***	102.9 ± 13.3	70.4 ± 2.7 **	535.7 ± 5.8	521.0 ± 1.5 **
HRT (ms)	72.1 ± 5.1	61.7 ± 3.1 *	97.6 ± 3.9	95.7 ± 1.7	90.4 ± 12.8	54.6 ± 2.4 **	138.5 ± 7.4	123.6 ± 2.6 *
Duration (ms)	324.3 ± 18.9	299.2 ± 12.0	720.0 ± 11.2	712.0 ± 5.1	389.2 ± 59.6	256.3 ± 11.9 *	853.2 ± 26.5	791.7 ± 9.1 *
CSA (mm^2^)	0.89 ± 0.06	0.90 ± 0.04	0.89 ± 0.06	0.90 ± 0.04	0.99 ± 0.09	1.03 ± 0.06	0.99 ± 0.09	1.03 ± 0.06
Fatigue at 50 (%)			33.6 ± 0.5	37.0 ± 3.0			46.1 ± 3.5	34.1 ± 2.6 *
Fatigue at 100 (%)			53.6 ± 0.6	59.8 ± 3.0			62.0 ± 2.9	51.7 ± 3.1 *
Fatigue at 150 (%)			62.3 ± 0.6	69.4 ± 2.9	14.0 ± 0.5	16.8 ± 0.6 **	68.3 ± 3.1	60.1 ± 3.3

Force parameters in the case of young animals from 9 (5 male and 4 female) and 10 (6 male and 4 female), in case of aged animals from 5 (2 male and 3 female) and 7 (5 male and 2 female) control and Syndecan-4 KD mice, respectively. *, **, and *** show significant difference from control at *p* < 0.05, *p* < 0.01, and *p* < 0.001, respectively.

**Table 5:** Parameters of voltage activation of calcium release in FDB.

	Young		Aged	
	CTRL	SDC4	CTRL	SDC4
V_50_ (mV)	−12.4 ± 2.3	−15.6 ± 3.2	−12.3 ± 3.3	−17.8 ± 2.5
k (mV)	6.7 ± 0.5	7.4 ± 1.2	9.2 ± 1.4	9.8 ± 1.0
Fitted [Ca^2+^]_i,max_ (F/F_0_)	1.99 ± 0.22	1.12 ± 0.16 **	2.04 ± 0.62	2.02 ± 0.36
Number of fibres	12	14	5	9

** shows a significant difference from the CTRL of the same age group at *p* < 0.01. The number and gender of mice are given in the legend of Figure 4.

## Data Availability

The datasets generated and analyzed during this study are available from the corresponding author upon reasonable request.

## Supplementary Materials

The following supporting information can be downloaded at: https://www.mdpi.com/article/10.3390/ijms24086933/s1.
