# Supplementary figures and images for: Unravelling the Effects of Syndecan-4 Knockdown on Skeletal Muscle Functions
Source: Int J Mol Sci. 2023 Apr 8;24(8):6933. doi: 10.3390/ijms24086933 (PMC10138797; doi:10.3390/ijms24086933)

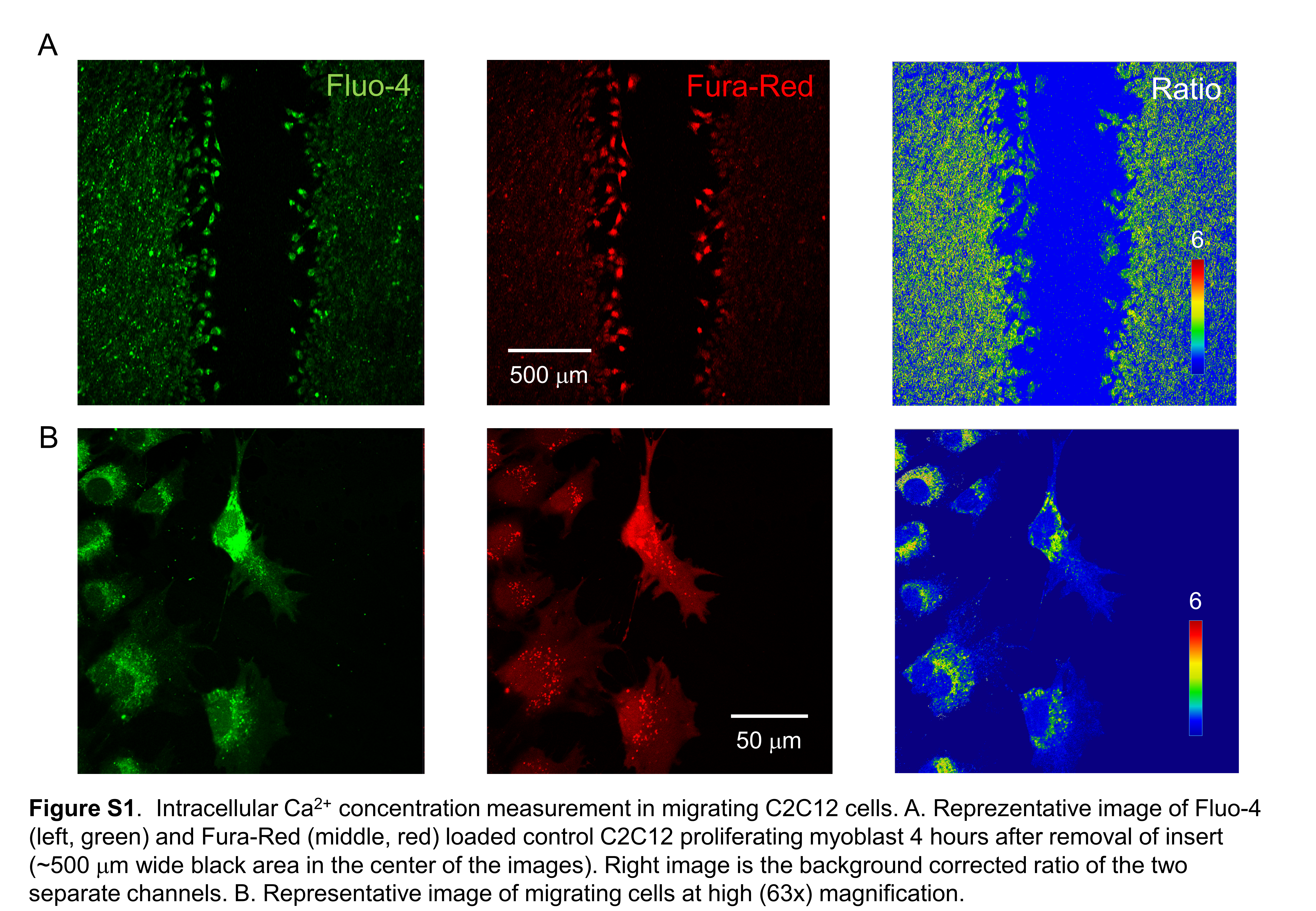

Supplement: Supplementary file 1 [file ijms-24-06933-s001.zip › Figure_S1.TIF]

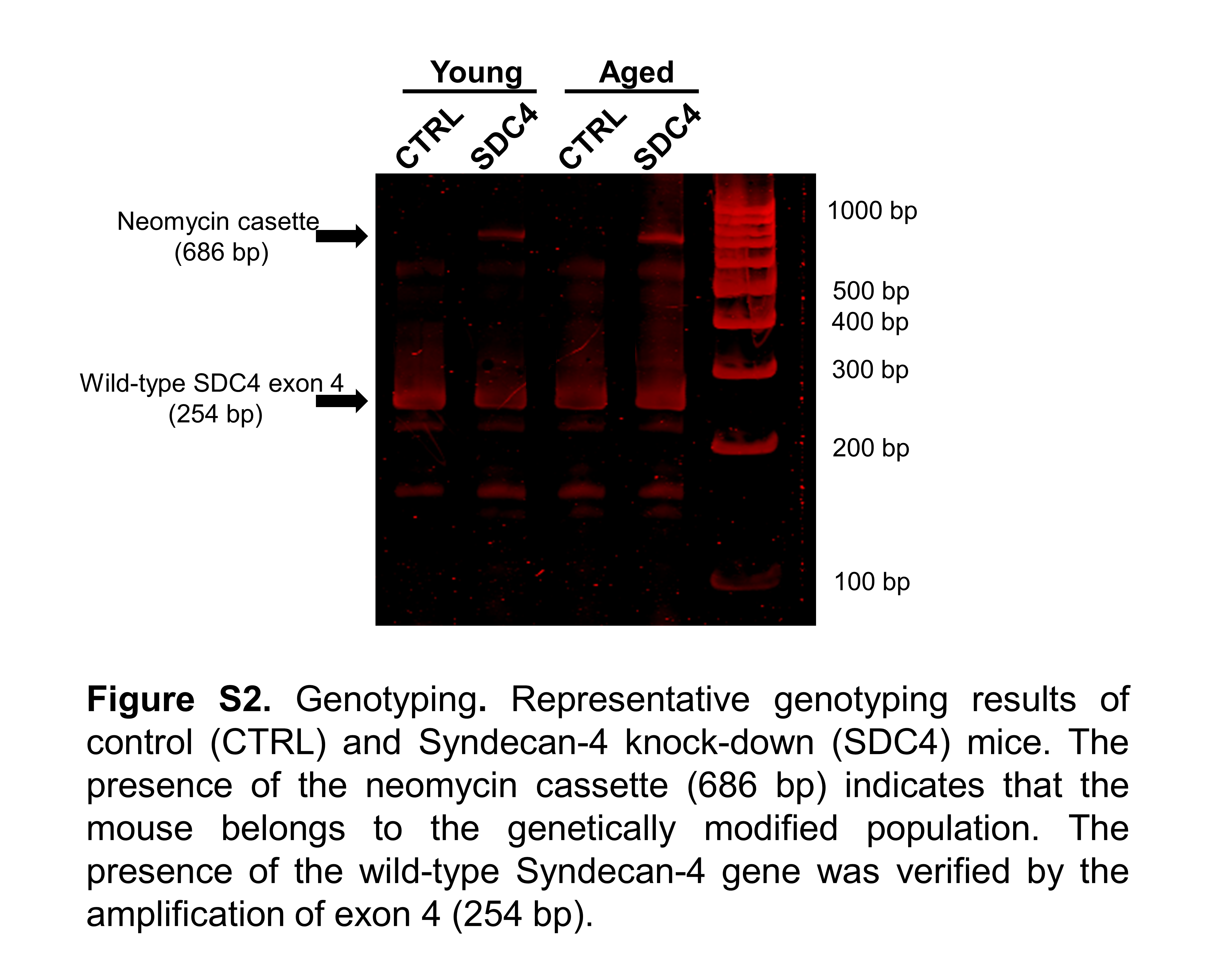

Supplement: Supplementary file 1 [file ijms-24-06933-s001.zip › Figure_S2.TIF]
